# Transition Metal Intercalators as Anticancer Agents—Recent Advances

**DOI:** 10.3390/ijms17111818

**Published:** 2016-10-31

**Authors:** Krishant M. Deo, Benjamin J. Pages, Dale L. Ang, Christopher P. Gordon, Janice R. Aldrich-Wright

**Affiliations:** 1 Nanoscale Organisation and Dynamics Group, Western Sydney University, Campbelltown, NSW 2560, Australia; K.Deo@westernsydney.edu.au (K.M.D.); B.Pages@westernsydney.edu.au (B.J.P.); D.Ang@westernsydney.edu.au (D.L.A.); 2 School of Science and Health, Western Sydney University, Campbelltown, NSW 2560, Australia; C.Gordon@westernsydney.edu.au

**Keywords:** cancer, intercalate, transition metals, DNA, cytotoxicity, DNA binding, platinum

## Abstract

The diverse anticancer utility of cisplatin has stimulated significant interest in the development of additional platinum-based therapies, resulting in several analogues receiving clinical approval worldwide. However, due to structural and mechanistic similarities, the effectiveness of platinum-based therapies is countered by severe side-effects, narrow spectrum of activity and the development of resistance. Nonetheless, metal complexes offer unique characteristics and exceptional versatility, with the ability to alter their pharmacology through facile modifications of geometry and coordination number. This has prompted the search for metal-based complexes with distinctly different structural motifs and non-covalent modes of binding with a primary aim of circumventing current clinical limitations. This review discusses recent advances in platinum and other transition metal-based complexes with mechanisms of action involving intercalation. This mode of DNA binding is distinct from cisplatin and its derivatives. The metals focused on in this review include Pt, Ru and Cu along with examples of Au, Ni, Zn and Fe complexes; these complexes are capable of DNA intercalation and are highly biologically active.

## 1. Introduction

**Figure 1:**
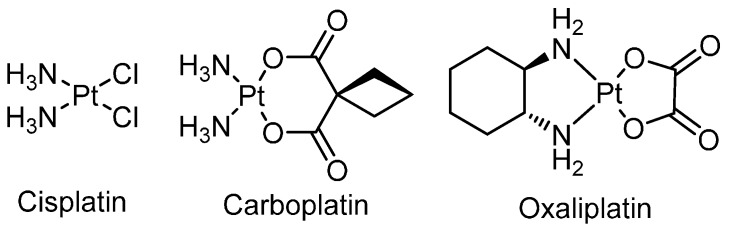
Chemical structures of cisplatin, carboplatin and oxaliplatin.

**Figure 2:**
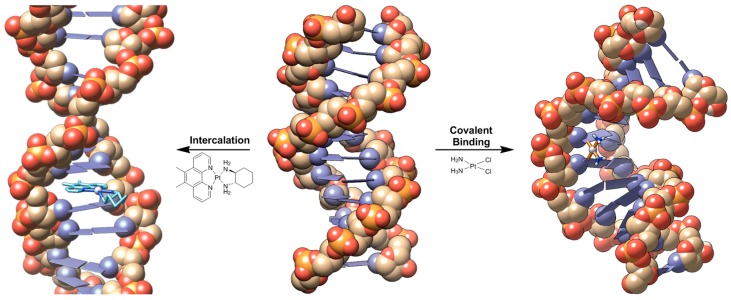
Schematic representation of a metal complex interacting with DNA, resulting in elongation of the double-helix (**left**, sourced from Protein Data Bank (PDB) file 2MG8 [9] with metal complex inserted manually) and cisplatin covalently binding to DNA, causing the double helix to bend (**right**, sourced from PDB 1AIO) [10]. Central DNA figure sourced from PDB file 1D86 [11]. Oxygen is orange, phosphorous is yellow, carbon is cream/white and nitrogen is blue/purple. The base pairs are also represented as blue/purple rectangular panels.

The anticancer activity of the platinum-based complex, cisplatin (Figure 1), was discovered in the 1960s and has since been used extensively for the treatment of various cancers including ovarian, testicular, lung and breast cancer [1,2,3]. This activity, however, is moderated by dose-limiting side-effects (nephro-, neuro- and ototoxicity) and development of resistance (acquired or intrinsic) [4]. In attempts to overcome the aforementioned, thousands of analogues have been synthesised, however, of these, only carboplatin (Figure 1) and oxaliplatin (Figure 1) have been approved for worldwide use. These complexes exhibit different side-effects and overcome some cisplatin resistance, respectively, although otherwise they demonstrate no significant improvements in efficacy overall [5,6]. This may be attributed to their similar geometrical configurations as they conform to the original structure-activity relationships that were reported requirements for exhibiting anticancer activity i.e., a neutral platinum(II) complex containing am(m)ine ligands and leaving group(s) that can be replaced during aquation [7,8]. The anticancer activity of cisplatin is generally attributed to its coordinative interaction with DNA. Upon entering the cell, the chloride ligands are substituted by water, forming strong electrophiles that can readily interact with nucleophilic bases of nucleic acids [6]. This results in the formation of covalently bound monofunctional and bifunctional adducts (mainly 1,2-intrastrand cross-links) that induce a conformational change to DNA which, through a series of events, ultimately leads to apoptosis (Figure 2) [6].

**Figure 3:**
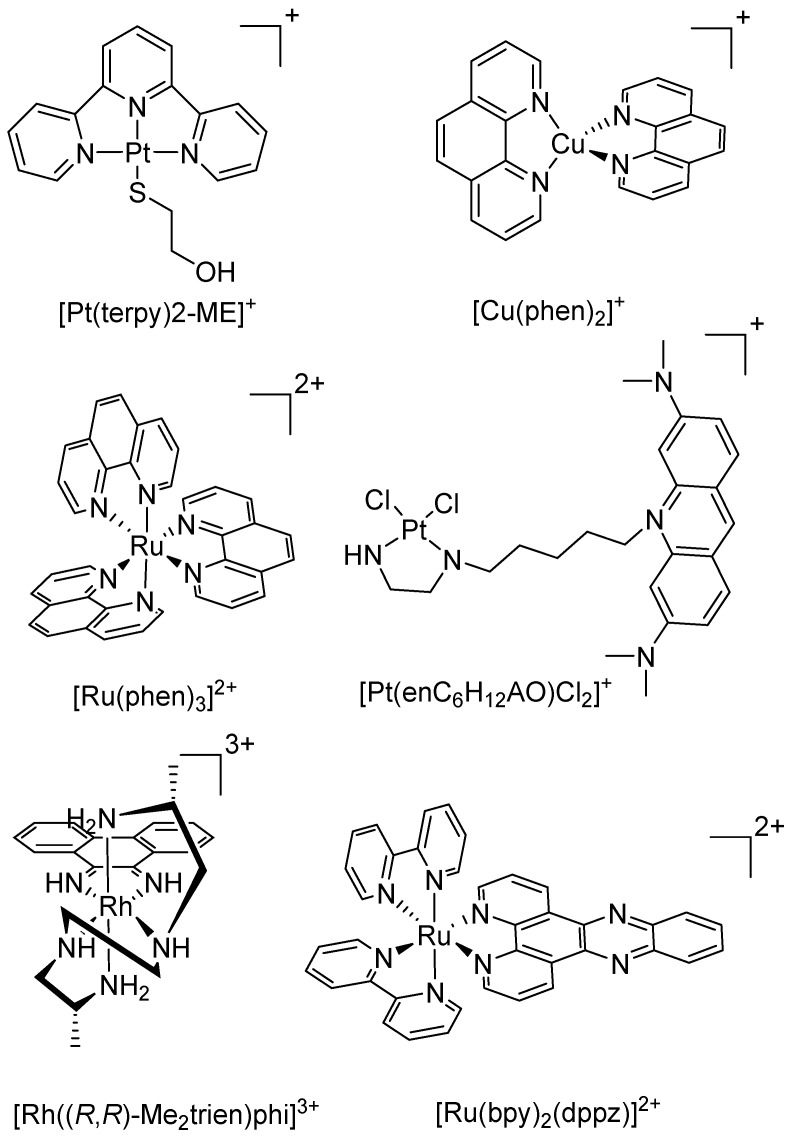
Chemical structures of early transition metal intercalators.

With the aim of developing metal-based complexes that exhibit improved pharmacological properties, efforts have been made to develop complexes with different modes of action and higher efficacy relative to cisplatin derivatives, including complexes that target cellular components other than DNA, complexes combined with delivery or targeting agents, or complexes that interact with DNA through non-covalent methods. A promising series of atypical anticancer metal complex are metallointercalators. Intercalation is the insertion of a complex within two adjacent base pairs of DNA [12]. Intercalators generally incorporate electron deficient, planar aromatic rings where non-covalent interactions with DNA are facilitated and stabilised through π–π stacking and dipole-dipole interactions, causing DNA to unwind and extend in order to accommodate the metal complex between the base pairs (Figure 2) [13,14]. This has the potential to circumvent recognition of repair mechanisms that lead to the resistance seen with cisplatin and its analogues. Transition metals deliver utility in anticancer drug design as they exhibit widely diverse geometries, coordination numbers, and selection of ligands that will coordinate, all with subtly different redox potentials and stabilities. Transition metal intercalators have been in development for decades; the original platinum complex [Pt(terpy)(2-ME)]^+^ (where 2-ME = 2-mercaptoethanol) was shown to bind strongly to DNA via intercalation, while a subsequent compound [Pt(enC_6_H_12_AO)Cl_2_]^+^ (where enC_6_H_12_AO is ethylenediamine tethered to acridine orange via an alkyl chain) bound with enhanced sequence-specificity for certain DNA sequences (Figure 3) [15,16]. The tetrahedral [Cu(phen)_2_]^+^ (where phen = 1,10-phenanthroline) was reported to be a potent inhibitor of polymerase I in *Escherichia coli*, which was achieved through DNA cleavage (Figure 3) [17]. The octahedral complex [Ru(phen)_3_]^2+^ is able to intercalate and unwind DNA as effectively as ethidium bromide while [Ru(bpy)_2_(dppz)]^2+^ (where bpy = 2,2’-bipyridine and dppz = dipyrido[3,2-*a*:2’,3’-*c*]phenazine) demonstrated enhanced luminescence upon intercalation with DNA, with the potential for use as a luminescent DNA probe (Figure 3) [18,19]. The rhodium complex, Δ-α-[Rh((*R*,*R*)-Me_2_trien)phi]^3+^ (where Me_2_trien = 2*R*,9*R*-diamino-4,7-diazadecane and phi = 9,10-phenanthrenequinone diamine), intercalates specifically to the major groove of the four base pair sequence 5′-TGCA-3′ and can also be photoactivated resulting in photoinduced oxidation of DNA (Figure 3) [20].

Due to their intrinsic properties, the impact of transition metals on the binding properties of the intercalating ligand can be staggering [21,22,23]; for example, a nickel complex of porphyrin was found to bind to DNA by intercalation, however the zinc complex of the same porphyrin could only bind through surface interactions due to the presence of an extra axial aqua ligand [24]. This review covers recent advances in anticancer intercalating complexes of a variety of transition metals. In particular, we focus on complexes that have had recent developments within the past three years, are confirmed to intercalate with DNA, and also exhibit high cytotoxcicty toward cancerous cells.

## 2. Platinum

**Figure 4:**
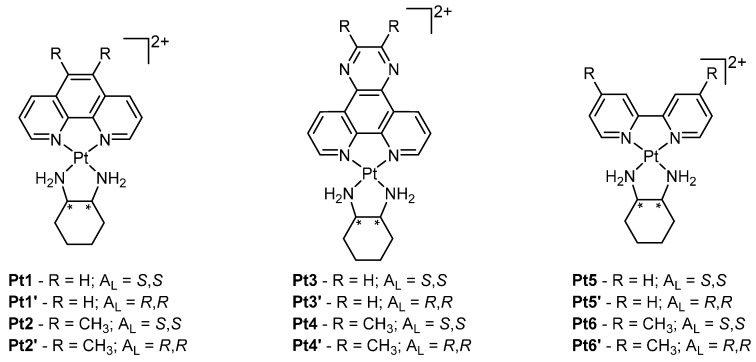
General structures of phen, dpq, bpy platinum intercalators. * indicates a stereocentre of the A_L_, either *S* or *R*. Counter ions have been omitted for clarity.

To date, the majority of platinum anticancer research has focused on the design of cisplatin analogues that covalently bind DNA. In contrast intercalators have received less attention; however, there are several recent examples of platinum intercalators that exhibit exceptionally high anticancer activity. A prominent series of complexes are composed of a general scaffold of [Pt(H_L_)(A_L_)]^2+^, where H_L_ is a heterocyclic intercalating ligand and A_L_ is a bidentate ancillary ligand [25,26]. These complexes include dipyrido[3,2-*f*:2’,3’-*h*]quinoxaline (dpq), 2,3-dimethyl-dpq (23Me_2_dpq), phen, 5,6-dimethyl-phen (56Me_2_phen), bpy or 4,4’-dimethyl-bpy (44Me_2_bpy) as the H_L_ and either the *S*,*S* or *R*,*R* isomer of 1,2-diaminocyclohexane (*S*,*S*- or *R*,*R*-dach) as the A_L_ (Figure 4).

Interactions of these platinum complexes (PCs) with DNA have been studied using various spectroscopic techniques as well as mass spectrometry and isothermal titration calorimetry, which provided evidence for a GC-selective intercalative binding mode and DNA affinity in the range of ~10^4^–10^6^ M^−1^ [27]. In vitro cytotoxicity assays showed greater activity than cisplatin and its analogues against a range of cell lines with a number of complexes demonstrating low-nanomolar activity (Table 1) [27]. For complexes consisting of bpy (i.e., **Pt5**), phen (i.e., **Pt1**) or their derivatives thereof, a correlation was apparent between DNA binding affinity and cytotoxicity where a higher DNA binding affinity was directly proportional to increased cytotoxicity, indicating DNA binding influences the apoptotic activity of these PCs. However, DNA affinity is not the only factor governing the activity of these complexes as the choice of A_L_ has a large effect. For example, complexes of *S*,*S*-dach (i.e., **Pt1** and **Pt3**) displayed higher cytotoxicity than those of *R*,*R*-dach (i.e., **Pt1’** and **Pt3’**), despite exhibiting the same DNA affinity [25].

The most promising analogue from this group of complexes is **Pt2** which exhibits over 160-fold greater activity than cisplatin in various cell lines (Table 1). To rationalise such a large difference in cytotoxicity, a comparative transcriptomics approach was undertaken between **Pt2** and cisplatin to distinguish the regulation of molecular pathways using the model organism *Saccharomyces cerevisiae* (yeast) [28,29]. Distinct differences were observed between treatment with **Pt2** and cisplatin at a molecular level, with stark contrasts in the up- and down-regulation of numerous molecular pathways. The sulphur-assimilation pathway was shown to be suppressed by **Pt2** while cisplatin caused an up-regulation of this pathway; this would subsequently result in increased production of thiol containing biomolecules such as thioredoxins and glutathione, which is thought to mediate resistance as it can deactivate PCs [29,30]. Additionally, genes regulating iron and copper transport across the cell membrane were significantly up-regulated by **Pt2** compared to cisplatin. The stark differences observed between **Pt2** and cisplatin in altering molecular pathways may partially account for their differences in cytotoxicity.

Despite **Pt2** exhibiting potent cytotoxicity in vitro, this activity has not yet translated into in vivo studies. BD-IX rats with peritoneal carcinomatosis (induced by intraperitoneal rat PROb colon cell inoculation) were treated with **Pt2**, via intravenous and intraperitoneal methods; this treatment did not elicit a tumour suppression response [32]. Furthermore, at pharmacological doses, **Pt2** seemed to cause nephrotoxicity [32]. However, in a separate study, the efficacy of **Pt1** was compared to cisplatin in female Specific Pathogen Free Swiss nude mice bearing PC3 (human prostate carcinoma) tumour xenografts [33]. Mice treated with either **Pt1** or cisplatin demonstrated a comparable decrease in mean tumour weight in relation to the control group. Furthermore, no obvious signs of toxicity were observed in mice treated with **Pt1**, while half of the cisplatin-treated mice perished by Day 20 [33].

**Figure 5:**
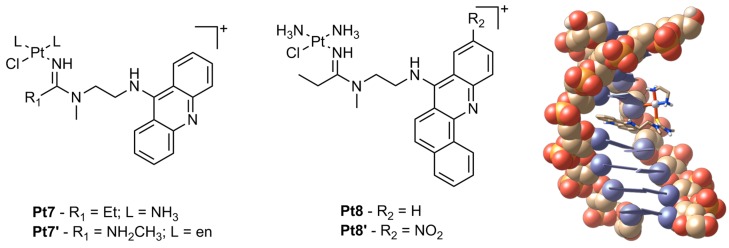
General structure of platinum complexes incorporating acridine and benz[c]acridine (left) and the acridine complex [PtCl(en)(1-{2-(acridin-9-ylamino)ethyl}-1,3-dimethylthiourea)](NO_3_)_2_ bound to DNA, as determined through a solution structure (PDB 1XRW) [39]. Counter-ions have been omitted for clarity and en = ethylenediamine. Oxygen is orange, phosphorous is yellow, carbon is cream/white and nitrogen is blue/purple. The base pairs are also represented as blue/purple rectangular panels.

Another promising class of platinum anticancer agents are composed of variations of *N*-[2-(acridin-9-ylamino)ethyl]-*N*-methylacetimidamide, linked by a chain of varying length to ligands conjugated around the platinum centre (Figure 5). These complexes have demonstrated exceptional cytotoxicity with **Pt7** exhibiting IC_50_ values down to nanomolar concentrations in non-small cell lung cancer (NSCLC) cell lines (Table 2) [34]. This activity has been attributed to the unique hybrid of DNA binding by these complexes which utilize both intercalation and nonfunctional adduct formation, which are more disruptive than those formed by cisplatin. The acridine moiety is able to intercalate whilst the platinum metal forms a monofunctional adduct with DNA adjacent to the intercalation site [35]. These lesions inhibit DNA synthesis through stalled replication forks and DNA double-strand breaks [35]. Furthermore, these adducts inhibit RNA polymerase II-mediated transcription more prominently than compared to cross-links by cisplatin [36].

Despite these platinum-acridine complexes exhibiting excellent in vitro cytotoxicity, in vivo studies have revealed severe unwanted toxicities in mice with xenografted NCI-H460 tumours. Although tumour growth was slowed, platinum levels were higher in healthy tissue than they were in the tumour, with the possibility of hepatotoxicity or nephrotoxicity as a result [37]. Hence variations of the original complex were synthesised in which the intercalating moiety was substituted with benz[c]acridine to increase its size and hydrophobicity (Figure 5, **Pt8**) [34]. **Pt8** was reported to be slightly less cytotoxic than **Pt7** (Table 2) although it was found to have significantly different cellular pharmacology and target binding properties, which may result in a more favourable therapeutic window in vivo.

**Figure 6:**
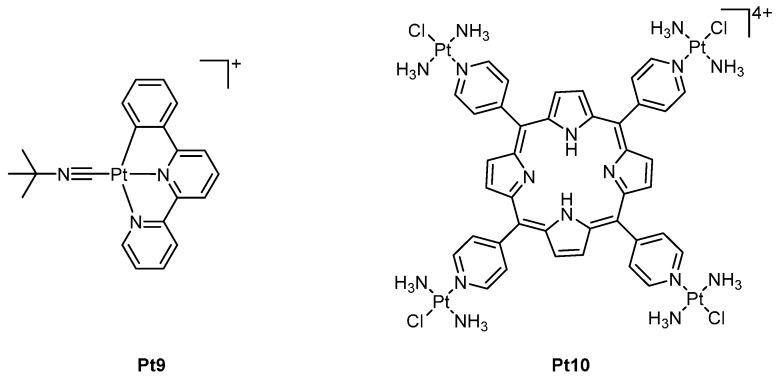
Structures of the luminescent cyclometalated PC, **Pt9** and the tetraplatinated porphyrin, **Pt10**. Counter-ions have been omitted for clarity.

More recently, a new class of luminescent cyclometalated PCs have been reported consisting of 6-phenyl-2,2’-bipyridyl (Figure 6). These complexes have demonstrated the ability to form emissive exciplexes with DNA via intercalation [40]. The most cytotoxic complex from this class of compounds, **Pt9**, exhibits in vitro IC_50_ values of 0.009 and 0.010 µM against oral epidermal carcinoma (KB) and neuroblastoma (SH-5YSY), respectively [40]. DNA damage by **Pt9**, resulting from its ability to stabilise the Topoisomerase I-DNA complex, has been attributed to its potent anticancer activity. Subsequent in vivo testing in nude mice implanted with NCI-460 showed tumour inhibition by 60% with no reported side-effects [40].

Platinated porphyrins are another class of complexes that have shown very promising results and have the potential to be activated via light irradiation (Figure 6). Fluorescence imaging experiments utilising the lead compound in this series **Pt10**, revealed the compound selectively localised within the nucleus. **Pt10** is reported to interact with DNA via a dual binding mode involving intercalation and, to a lesser extent, covalent binding through the platinum centres [41]. DNA photocleavage experiments showed no damage to DNA in the absence of light, however upon irradiation, photocleavage of DNA was markedly enhanced [41]. In vitro studies of **Pt10** in cisplatin-resistant human ovarian cancer (CP70) cell lines showed IC_50_ values of >100 µM in the absence of light, however, when irradiated (420 nm, 6.95 J·cm^−2^), this IC_50_ value decreased significantly to 0.019 µM [41].

## 3. Copper

**Figure 7:**
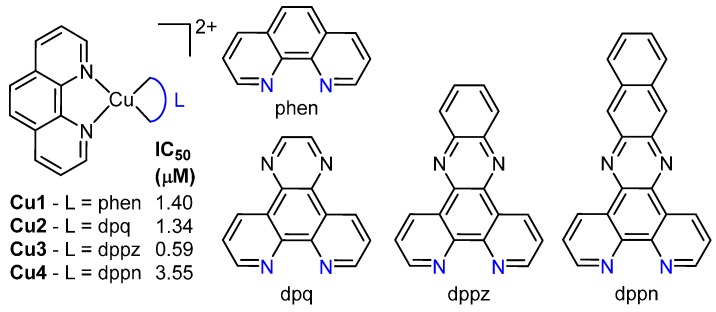
Structures of copper complexes **Cu1**–**4**, and the IC_50_ value of each complex in the SKOV3 human cancer cell line. Blue-coloured atoms are those that coordinate to the copper centre for each L example. Counter-ions have been omitted for clarity.

Copper has a long history in medicinal inorganic chemistry, particularly in antibacterial and anticancer agents, due to its natural bioavailability, its role in angiogenesis and increased uptake in cancerous tissues [42,43]. The role of copper in the growth of tumours is significant enough that copper capturing agents have progressed to phase II clinical trials [44,45]. Copper complexes most often initiate their cytotoxic effect through oxygen-dependent or -independent DNA cleavage. Here, DNA intercalation can assist the cleavage process by allowing close proximity of copper complexes to the double strand [46,47,48]. Copper is also capable of a large variety of coordination geometries, often producing very different species from reactions with very similar starting reagents[46,47]. The most well-known types of copper nucleases are those incorporating phen such as [Cu(phen)_2_]^2+^. In a recent study, this scaffold was modified to afford additional complexes of the type [Cu(phen)(L)], where L is one of phen (**Cu1**), dpq (**Cu2**), dppz (**Cu3**) or benzo[i]dppz (dppn, **Cu4**, Figure 7) [49]. Intercalation was theorised to occur within both the major and minor grooves; **Cu2** and **Cu3** demonstrated 60-hold higher CT-DNA binding affinity than **Cu1** with binding constants of approximately 3 × 10^7^ M. All complexes could cleave plasmid DNA through an oxidative mechanism, and each exhibited low-micromolar activity against SKOV3 human cancer cells (Figure 7).

**Figure 8:**
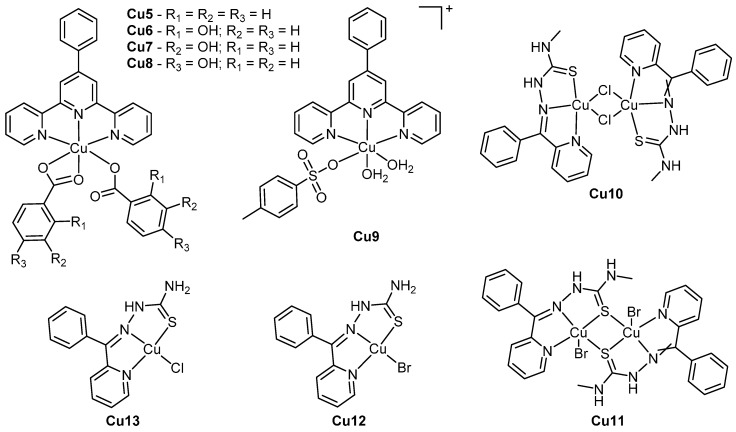
Structures of complexes **Cu5**–**13**. Counter-ions have been omitted for clarity.

The scope of copper nucleases expands far beyond copper phenanthrenes. For example a recent series of complexes of the type [Cu(4phterpy)(L)_2_] or [Cu(4phterpy)(L)(H_2_O)_2_](L) (where 4phterpy is 4’-phenyl-2,2’:6’,2”-terpyridine and L is one of *p*-toluenesulphonate, benzoate or *o*-, *m*- or *p*-hydroxybenzoate, **Cu5**–**9**, Figure 8) were found to be cytotoxic to HCT116 colorectal carcinoma and HepG2 hepatocellular carcinoma cells while exhibiting lower activity in normal human fibroblasts (Table 3) [48]. The model complex **Cu9** induced apoptosis in HCT116 cells in a caspase-3 related mechanism; the higher cytotoxicity of **Cu9** relative to the others was theorised to be due to its labile aqua ligands and charged nature, which could encourage cellular uptake by human copper transporters [50]. All complexes intercalated with DNA, exhibiting binding constants of 10^5^–10^6^ M^−1^, and each was capable of hydrolytically cleaving plasmid DNA under both aerobic and anaerobic conditions in a radical and oxygen-independent manner. **Cu6** produced a substantial amount of linear DNA during cleavage experiments relative to **Cu5**, **Cu7** and **Cu8**, suggesting that the *ortho* position of the benzoate hydroxyl group was optimal for DNA cleavage.

Another recent study focused on a series of copper semicarbazone complexes: [Cu(Bp4mT)(μ-Cl)]_2_ (**Cu10**), [Cu(μ-Bp4mT)Br]_2_ (**Cu11**), [Cu(HBpT)Cl] (**Cu12**), and [Cu(HBpT)Br] (**Cu13**) (where Bp4mT is 2-benzoylpyridine-4-methylthiosemicarbazone and HBpT is 2-benzoylpyridinethiosemicarbazone, (Figure 8) [47]. Each complex is capable of intercalation and DNA cleavage through an oxidative mechanism involving hydroxide radicals and singlet oxygen. All complexes were found to be at least ten times more active than cisplatin against HeLa, HepG-2 and NCI-H460 cells, achieving IC_50_ values as low as 0.08 ± 0.01 μM (Table 3). The dinuclear complexes **Cu10** and **Cu11** were more than twice as active as the mononuclear **Cu12** and **Cu13**; and it was proposed that the increased lipophilicity afforded by the methylated nitrogen of the Bp4mT ligand could increase the passive diffusion of **Cu10** and **Cu11** into cancerous cells. Alternatively, it could also be as a consequence of double the quantity of active components in the dimer.

There are many other notable recent studies of intercalating copper nucleases [46,51,52,53,54]. Two copper intercalators of (2-((quinolin-8-ylimino)methyl)pyridine) recently exhibited activity against HeLa, MCF-7 and A549 cells and cleaved DNA without addition of peroxide [46]. A *bis*-thiosemicarbazone copper complex was found to intercalate and cleave DNA, exhibit micromolar-level activity against HCT116 cells, and induce augmented tumour regression in a murine HCT116 cell xenograft model. However, the DNA binding and biological activity were not necessarily correlated for all complexes in the study [51]. Overall, copper intercalators have demonstrated potential as anticancer agents due to their efficient DNA binding and cleavage activity.

## 4. Ruthenium

**Figure 9:**
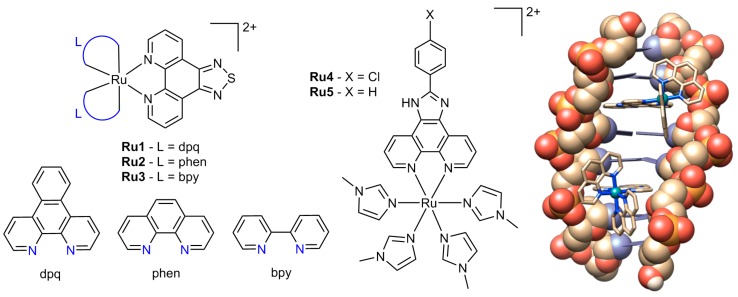
Chemical structures of ruthenium polypyridyl complexes **Ru1**–**5** (**left**) and the X-ray crystal structure of *rac*-[Ru(phen)_2_(dppz)]^2+^ bound to DNA sequence d(ATGCAT)_2_ (**right**). The extended aromatic ligand intercalates and separates the DNA base pairs, here shown with both the ∆ and Λ enantiomers bound. Sourced from PDB file 4JD8 [59]. Blue-coloured atoms are those that coordinate to the ruthenium centre in each L example. Counter-ions have been omitted for clarity. Oxygen is orange, phosphorous is yellow, carbon is cream/white and nitrogen is blue/purple. The base pairs are also represented as blue/purple rectangular panels.

The octahedral geometry and interchangeable oxidation states of Ru(II) and Ru(III) allow for a large diversity of ligand combinations, and the inherent fluorescent properties and high kinetic stability of ruthenium compounds are extremely beneficial to biological studies [55], as well as the design of photo-activated complexes [56]. Some ruthenium anticancer complexes have advanced to clinical trials, with the complex indazolium *trans*-[tetrachlorobis(1*H*-indazole)ruthenate(III)] (KP1019) successfully completing phase I clinical trials [57], whereas the complex (ImH)[*trans*-RuCl_4_(DMSO)(Im)] (NAMI-A, where Im = imidazole, DMSO = dimethylsulphoxide) has progressed to phase II clinical trials [58]. The most common type of ruthenium intercalators are polypyridyl complexes [Ru(L_1_)_2_(L_2_)]^2+^, in which L_2_ is a long intercalating ligand such as dppz or dppn and L_1_ are two ancillary ligands that can affect DNA binding properties (Figure 6) [59]. While these types of complexes often display low levels of cytotoxicity, there are several polypyridyl compounds recently synthesised that demonstrate potent activity. A recent series of complexes of the type [Ru(L)_2_(tdzp)]^2+^ (where L is one of bpy, phen or dpq and tdzp is [1,2,5]-thiadiazolo-[3,4-*f*]-[1,10]-phenanthroline, **Ru1**–**3**, Figure 9) were found to intercalate with DNA with binding constants of 10^3^–10^4^ M^−1^ in a manner dependent on the planarity of ligand. These complexes accumulated within the nuclei of cells and were antiproliferative against HeLa cells (Table 4) [60]. In a different study, complexes of the type [Ru(MeIm)_4_(L)]^2+^ (where MeIm = 1-methylimidazol and L = 2-(4-chlorophenyl)-1*H*-imidazo[4,5-*f*] [1,10]phenanthroline, **Ru4**, or 2-phenyl-1*H*-imidazo[4,5-*f*] [1,10]phenanthroline, **Ru5**, Figure 9) were shown to intercalate with DNA and cause cell cycle arrest in the A549 cell line at G_0_/G_1_ phase.The complexes also induced mitochondrial dysfunction and ultimately apoptosis involving ROS accumulation and Bcl-2 and caspase family activation. The cytotoxicity of each complex was comparable with cisplatin in several cell lines, with **Ru4** being more active than **Ru5** (Table 4) [61].

**Figure 10:**
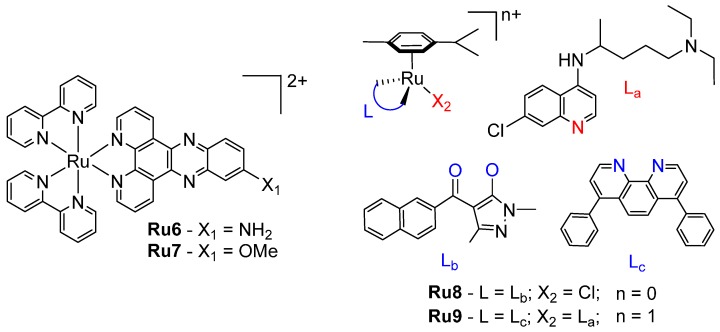
Chemical structures of ruthenium arene complexes **Ru6**–**9**. Counter-ions have been omitted for clarity. Ligands with a blue label coordinate at the “L” position of the arene through the blue-coloured oxygen or nitrogen atoms. L_a_ coordinates at the X_2_ position through the red nitrogen.

Polypyridyl ruthenium complexes have also been used as intercalating photodynamic agents [63,64]. Complexes of the type [Ru(bpy)_2_(R-dppz)]^2+^ (where R is either NH_2_, **Ru6** or OMe, **Ru7**, Figure 10) intercalated with DNA via the R-dppz ligand and achieved phototoxic indices of >150 and 42, respectively, against HeLa cells when irradiated with light at 420 nm (Table 4) [62]. A polypyridyl ruthenium complex incorporating an appended anthracene demonstrated photocleavage of DNA through both ruthenium-derived singlet oxygen and anthracene-derived radicals, as well as light-induced cytotoxicity in F98 glioma cells [65,66]. Ruthenium arene “piano-stool” complexes are very prominent anticancer agents, and some studies have reported a dual-binding mode in which ruthenation occurs through a leaving group while the *p*-cymene group intercalates between nearby bases [67]. Recent examples include a series of complexes incorporating 1,3-Dimethyl-4-acylpyrazolon-5-ato ligands, of which the lead compound, [Ru(*p*-cymene)(1,3-dimethyl-4-(1-naphthoyl)-pyrazolon-5-ate)Cl] (**Ru8**, Figure 10), demonstrated potent activity against several cell lines [67], and a chloroquine-tethered complex with submicromolar activity against A549 and MCF-7 cells (**Ru9**, Figure 10) [68].

## 5. Other Metals

**Figure 11:**
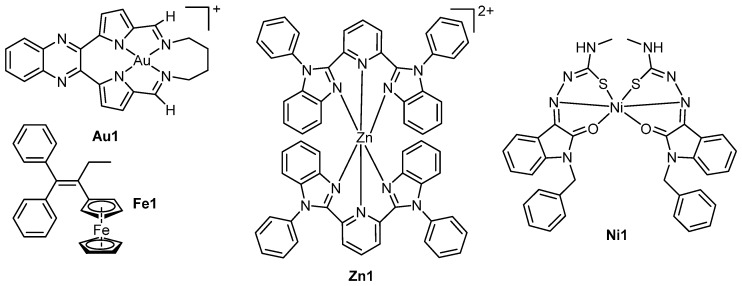
Structures of the metallointercalators **Au1**, **Fe1**, **Zn1** and **Ni1**. Counter-ions have been omitted for clarity.

A large spectrum of transition metal complexes have been used as anticancer agents, although for many intercalation is not required for activity. Nonetheless some recent examples of less common transition metal intercalators have emerged in the literature. For example, gold complexes have been relatively successful in medicinal chemistry [69,70,71], although most active anticancer complexes do not target DNA [72,73,74]. Recently reported macrocyclic gold(III) complexes that incorporated a quinoxaline moiety to promote DNA intercalation demonstrated cytotoxic activity [75]. The lead compound, [Au(12,13,14,15-tetrahydro-6,9:18,21-diepimino[1,6]diazacycloctadecino[12,13-*b*]quinoxaline)]^+^ (**Au1**, Figure 11) exhibited low micromolar activity in a panel of human cell lines, particularly in leukaemia and central nervous system cancers, and was well-tolerated by nude tumour-less mice at high doses. Enzyme inhibition assays, molecular modelling and surface plasmon resonance studies revealed that **Au1** was an inhibitor of human topoisomerase 1 (Top1). Here inhibition occurred through intercalative binding to the DNA substrate of Top1 and not through binding to Top1 itself. Another study focused on gold(I) complexes consisting of a DNA intercalating 1,8-naphthalimide tethered to a gold centre through an *N*-heterocyclic carbene moiety [76]. The complexes were designed as dual-action anticancer agents that both intercalate with DNA and inhibit thioredoxin reductase activity; low micromolar activity was exhibited by the four complexes in HT-29 and MCF-7 cell lines.

Nickel and zinc have also seen widespread use in medicinal chemistry as DNA nucleases due to their natural abundance in humans and important roles in cellular functions [77,78]. A recent zinc study focused upon complexes of 2,6-*bis*(1-phenyl-1*H*-benzo[d]imidazol-2-yl)pyridine (bpbp) [77]. The lead compound [Zn(bpbp)_2_]^2+^ (**Zn1**, Figure 11) demonstrated low micromolar activity in a variety of cell lines, with in IC_50_ of 2.9 ± 0.3 μM against MCF-7 cells. The hypothesised mechanism of action was DNA damage via intercalation and cleavage, resulting in elevated levels of phosphorylated p53 gene and apoptosis. Another intercalating zinc complex of 5-bromo-8-hydroxyquinoline displayed higher cytotoxicity in BEL-7404 and T-24 cells than cisplatin and induced cell cycle arrest in the G_2_ phase of the BEL-7404 cells [79]. A series of nickel isotin thiosemicarbazone complexes were also found to intercalate with DNA and achieve up to 99.8% cleavage of plasmid DNA without the addition of peroxide [78]. The lead compound, [Ni(L)_2_] (where L = (*Z*)-2-(1-benzyl-2-oxoindolin-3-ylidene)-*N*-methylhydrazinecarbothioamide, **Ni1**, Figure 11) was highly active against MCF7 cells with an IC_50_ value of <0.1 μM. In an additional study, nickel and cobalt complexes of the anaesthetic lidocaine produced low micromolar IC_50_ values against a panel of human cell lines. Here the nickel complexes were generally more active than the cobalt [80]. All complexes in the study cleaved plasmid DNA in the presence of H_2_O_2_ in a singlet oxygen-involved method.

Iron has also been exploited in the development of anticancer agents as it is an essential component of various biological processes including erythropoiesis, electron transport and DNA synthesis [81]. Additionally, iron most commonly exists in two oxidation states (Fe(II) and (Fe(III)), which allows it to participate in important redox reactions [82]. A recent series of iron-based complexes that incorporate ferrocene as part of a modified tamoxifen base structure have been reported (Figure 11). The lead compound, **Fe1**, showed no activity against non-cancerous glomerular basement membrane (GBM) monkey cells, however exhibited low micromolar activity in cancerous cell lines with IC_50_ values of 0.9 and 1.04 µM against human colon cancer (HCT-8) and acute promyelocytic leukaemia (HL-60), respectively [83]. Mechanistic studies revealed the activation of caspases 3 and 7, externalisation of phosphatidylserine and increased DNA fragmentation, which were attributed to an intercalative mode of binding of **Fe1** with dsDNA and ssDNA [83].

## 6. Conclusions

Over the past five decades, an extensive catalogue of metal complexes have been synthesised and evaluated as potential anticancer agents, with the primary aim of overcoming the drawbacks of currently used metallodrugs. Transition metal intercalators have been studied since the 1970s as alternatives that exerts cytotoxicity through different modes of action to cisplatin-like chemotherapeutics. A variety of transition metal-based intercalators have been reviewed here, all of which are potently cytotoxic and demonstrate DNA intercalation. Platinum intercalators can kill cancerous cells via unconventional methods at nanomolar concentrations, and some have also demonstrated some tumour-inhibition results in vivo. Copper complexes can intercalate and cleave DNA, with recent studies reporting potent compounds both with the phenanthrene architype coordination and without. Nickel and zinc intercalators are also proven to be efficient DNA nucleases. Ruthenium polypyridyl and arene complexes have exhibited micromolar activity against several human cell lines and some have proven potential as photoactivated drugs. Recent pairing of intercalating ligands with other metals such as gold and iron has produced even more cytotoxic complexes with distinctly different mechanisms to kill cancerous cells. The huge variety of transition metal properties and ligand combinations has produced an extremely broad spectrum of intercalating anticancer complexes, each with a unique mechanism of action. The continued expansion of this spectrum has great potential to reveal metallointercalators which can outperform current metallodrugs and provide more effective chemotherapy.

## Figures and Tables

**Table 1:** In vitro cytotoxicity of **Pt1**–**6** and **Pt1’**–**6’** against L1210 (murine leukaemia) and Du145 (prostate cancer) and A2780 (human ovarian cancer) cell lines. IC_50_ is the concentration at which cell growth is inhibited by 50% over 72 h. Values taken from reference [27].

Complex	IC_50_ (µM)		
	L1210	Du145	A2780
**Pt1**	0.10 ± 0.01	0.08 ± 0.05	0.27 ± 0.03
**Pt1’**	1.5 ± 0.1	0.79 ± 0.08	2.7 ± 0.07
**Pt2**	0.009 ± 0.002	0.007 ± 0.002	0.030 ± 0.004
**Pt2’**	0.46 ± 0.01	0.41 ± 0.04	1.1 ± 0.1
**Pt3**	0.19 ± 0.01	0.44 ± 0.06	2.0 ± 0.1
**Pt3′**	0.8 ± 0.2	2.7 ± 0.2	6.5 ± 0.0
**Pt4**	1.3 ± 0.4	2.2 ± 0.1	3.7 ± 0.4
**Pt4′**	6 ± 2	3 ± 1	2.0 ± 0.1
**Pt5**	0.6 ± 0.2	1.3 ± 0.4	2.6 ± 0.2
**Pt5′**	5.5 ± 0.1	n.d.	n.d
**Pt6**	0.36 ± 0.02	0.12 ± 0.03	1.1 ± 0.3
**Pt6’**	1.8 ± 0.0	1.5 ± 0.03	5.6 ± 0.5
Cisplatin	0.35–1 ^[a]^	1.2 ± 0.1	1.0 ± 0.1
Carboplatin	n.d.	2.9 ± 0.4	0.16 ± 0.0
Oxaliplatin	n.d.	15 ± 1	9 ± 3

^[a]^ Value obtained from references [25,31]. n.d. = not determined.

**Table 2:** In vitro cytotoxicity of **Pt7**, **Pt7’**, **Pt8** and **Pt8’** against human NSCLC cell lines and HL-60 leukaemia cells. Values taken from references [34,38].

Complex	IC_50_ (µM)				
	Cell Line				
	NCI-H460	NCI-H520	NCI-H522	A549	HL-60
**Pt7**	0.0052 ± 0.0001	0.043 ± 0.004	0.010 ± 0.001	0.0065 ± 0.0002	–
**Pt7’**	–	–	–	–	0.13
**Pt8**	0.24 ± 0.01	0.52 ± 0.01	0.12 ± 0.02	0.32 ± 0.06	–
**Pt8’**	2.4 ± 0.5	2.2 ± 0.1	3.62 ± 0.08	12.4 ± 0.9	–

**Table 3:** In vitro cytotoxicity of complexes **Cu5**–**13** in various cell lines, expressed as IC_50_ values with standard error (1 significant figure).

Complex	IC_50_ (μM)			Reference
	HCT116	HepG-2	NHF ^[a]^	
**Cu5**	0.31 ± 0.03	14.0 ± 0.5	>20	[48]
**Cu6**	0.468 ± 0.006	13.6 ± 0.5	>20	
**Cu7**	0.44 ± 0.09	0.54 ± 0.03	>5	
**Cu8**	1.5 ± 0.2	0.7 ± 0.1	>5	
**Cu9**	0.07 ± 0.05	0.24 ± 0.02	5.483 ± 0.003	
Complex	HeLa	HepG-2	NCI-H460	Reference
**Cu10**	0.16 ± 0.05	0.10 ± 0.04	0.08 ± 0.01	[47]
**Cu11**	0.59 ± 0.02	0.20 ± 0.01	0.16 ± 0.01	
**Cu12**	1.4 ± 0.6	1.1 ± 0.4	2.0 ± 0.3	
**Cu13**	1.3 ± 0.2	0.8 ± 0.2	1.5 ± 0.7	

^[a]^ NHF = normal human fibroblasts.

**Table 4:** In vitro cytotoxicity of complexes **Ru1–7** in HeLa cells, expressed as IC_50_ values with standard error (1significant figure). Cisplatin is included as a reference.

Complex	IC_50_ (µM)	Reference	Complex	IC_50_ (µM)	Reference
**Ru1**	28.0 ± 0.1	[60]	**Ru6** ^[a]^	2.0 ± 0.9	[62]
**Ru2**	21.00 ± 0.08		**Ru7** ^[a]^	5.5 ± 0.7	
**Ru3**	19.00 ± 0.08		–	–	–
**Ru4**	27 ± 2	[61]	**Cisplatin**	15 ± 2	[61]
**Ru5**	25 ± 2				

^[a]^ Values account for irradiation at 420 nm.

## References

[1] Rosenberg B., van Camp L., Krigas T. (1965). Inhibition of cell division in *Escherichia coli* by electrolysis products from a platinum electrode. Nature.

[2] Loehrer P.J., Einhorn L.H. (1984). Drugs five years later. Cisplatin. Ann. Intern. Med..

[3] Provencher-Mandeville J., Debnath C., Mandal S.K., Leblanc V., Parent S., Asselin É., Bérubé G. (2011). Design, synthesis and biological evaluation of estradiol-PEG-linked Platinum(II) hybrid molecules: Comparative molecular modeling study of three distinct families of hybrids. Steroids.

[4] Cepeda V., Fuertes M.A., Castilla J., Alonso C., Quevedo C., Pérez J.M. (2007). Biochemical mechanisms of cisplatin cytotoxicity. Anti-Cancer Agents Med. Chem..

[5] Florea A.M., Büsselberg D. (2011). Cisplatin as an anti-tumor drug: Cellular mechanisms of activity, drug resistance and induced side effects. Cancers.

[6] Johnstone T.C., Wilson J.J., Lippard S.J. (2013). Monofunctional and higher-valent platinum anticancer agents. Inorg. Chem..

[7] Cleare M.J., Hoeschele J.D. (1973). Studies on the antitumor activity of group VIII transition metal complexes. Part I. Platinum (II) complexes. Bioinorg. Chem..

[8] Lovejoy K.S., Lippard S.J. (2009). Non-traditional platinum compounds for improved accumulation, oral bioavailability, and tumor targeting. Dalton Trans..

[9] Lin C., Mathad R.I., Zhang Z., Sidell N., Yang D. (2014). Solution structure of a 2:1 complex of anticancer drug XR5944 with TFF1 estrogen response element: Insights into DNA recognition by a bis-intercalator. Nucleic Acids Res..

[10] Gelasco A., Lippard S.J. (1998). NMR solution structure of a DNA dodecamer duplex containing a *cis*-Diammineplatinum(II) d(GpG) intrastrand cross-link, the major adduct of the anticancer drug cisplatin. Biochemistry.

[11] Drew H.R., Wing R.M., Takano T., Broka C., Tanaka S., Itakura K., Dickerson R.E. (1981). Structure of a B-DNA dodecamer: Conformation and dynamics. Proc. Natl. Acad. Sci. USA.

[12] Lerman L.S. (1961). Structural considerations in the interaction of DNA and acridines. J. Mol. Biol..

[13] Long E.C., Barton J.K. (1990). On demonstrating DNA intercalation. Acc. Chem. Res..

[14] Garbutcheon-Singh K.B., Myers S., Harper B.W.J., Ng N.S., Dong Q., Xie C., Aldrich-Wright J.R. (2013). The effects of 56MESS on mitochondrial and cytoskeletal proteins and the cell cycle in MDCK cells. Metallomics.

[15] Jennette K.W., Lippard S.J., Vassiliades G.A., Bauer W.R. (1974). Metallointercalation reagents. 2-hydroxyethanethiolato(2,2’,2’-terpyridine)-platinum(II) monocation binds strongly to DNA by intercalation. Proc. Natl. Acad. Sci. USA.

[16] Bowler B.E., Lippard S.J. (1986). Modulation of platinum antitumor drug binding to DNA by linked and free intercalators. Biochemistry.

[17] Sigman D.S., Graham D.R., D’Aurora V., Stern A.M. (1979). Oxygen-dependent cleavage of DNA by the 1,10-phenanthroline. Cuprous complex. Inhibition of *Escherichia coli* DNA polymerase I. J. Biol. Chem..

[18] Kelly J.M., Tossi A.B., McConnell D.J., OhUigin C. (1985). A Study of the interactions of some Polypyridylruthenium(II) complexes with DNA using fluorescence spectroscopy, topoisomerisation and thermal denaturation. Nucleic Acids Res..

[19] Friedman A.E., Chambron J.C., Sauvage J.P., Turro N.J., Barton J.K. (1990). A molecular light switch for DNA: Ru(bpy)_2_(dppz)^2+^. J. Am. Chem. Soc..

[20] Kielkopf C.L., Erkkila K.E., Hudson B.P., Barton J.K., Rees D.C. (2000). Structure of a photoactive rhodium complex intercalated into DNA. Nat. Struct. Mol. Biol..

[21] Yan Y.K., Melchart M., Habtemariam A., Sadler P.J. (2005). Organometallic chemistry, biology and medicine: Ruthenium arene anticancer complexes. Chem. Commun..

[22] Cohen S.M. (2007). New approaches for medicinal applications of bioinorganic chemistry. Curr. Opin. Chem. Biol..

[23] Romero-Canelón I., Sadler P.J. (2013). Next-generation metal anticancer complexes: Multitargeting via redox modulation. Inorg. Chem..

[24] Asadi M., Safaei E., Ranjbar B., Hasani L. (2004). Thermodynamic and spectroscopic study on the binding of cationic Zn(II) and Co(II) tetrapyridinoporphyrazines to calf thymus DNA: The role of the central metal in binding parameters. New J. Chem..

[25] Pages B.J., Li F., Wormell P., Ang D.L., Clegg J.K., Kepert C.J., Spare L.K., Danchaiwijit S., Aldrich-Wright J.R. (2014). Synthesis and analysis of the anticancer activity of Platinum(II) complexes incorporating dipyridoquinoxaline variants. Dalton Trans..

[26] Pages B.J., Zhang Y., Li F., Sakoff J., Gilbert J., Aldrich-Wright J.R. (2015). Cytotoxicity and structural analyses of 2,2′-Bipyridine-, 4,4′-Dimethyl-2,2′-bipyridine- and 2-(2′-Pyridyl)quinoxalineplatinum(II) complexes. Eur. J. Inorg. Chem..

[27] Pages B.J., Sakoff J., Gilbert J., Rodger A., Chmel N.P., Jones N.C., Kelly S.M., Ang D.L., Aldrich-Wright J.R. (2016). Multifaceted studies of the DNA interactions and in vitro cytotoxicity of anticancer polyaromatic Platinum(II) complexes. Chem. Eur. J..

[28] Wang S., Higgins V., Aldrich-Wright J., Wu M. (2012). Identification of the molecular mechanisms underlying the cytotoxic action of a potent platinum metallointercalator. J. Chem. Biol..

[29] Wang S., Wu M.J., Higgins V.J., Aldrich-Wright J.R. (2012). Comparative analyses of cytotoxicity and molecular mechanisms between platinum metallointercalators and cisplatin. Metallomics.

[30] Kemp S., Wheate N.J., Pisani M.J., Aldrich-Wright J.R. (2008). Degradation of bidentate-coordinated platinum(II)-based DNA intercalators by reduced l-glutathione. J. Med. Chem..

[31] Wheate N.J., Taleb R.I., Krause-Heuer A.M., Cook R.L., Wang S., Higgins V.J., Aldrich-Wright J.R. (2007). Novel Platinum(II)-based anticancer complexes and molecular hosts as their drug delivery vehicles. Dalton Trans..

[32] Moretto J., Chauffert B., Ghiringhelli F., Aldrich-Wright J.R., Bouyer F. (2011). Discrepancy between in vitro and in vivo antitumor effect of a new Platinum(II) metallointercalator. Investig. New Drug..

[33] Fisher D.M., Fenton R.R., Aldrich-Wright J.R. (2008). In vivo studies of a Platinum(II) metallointercalator. Chem. Commun..

[34] Pickard A.J., Liu F., Bartenstein T.F., Haines L.G., Levine K.E., Kucera G.L., Bierbach U. (2014). Redesigning the DNA-targeted chromophore in platinum–acridine anticancer agents: A structure-activity relationship study. Chem. Eur. J..

[35] Cheung-Ong K., Song K.T., Ma Z., Shabtai D., Lee A.Y., Gallo D., Heisler L.E., Brown G.W., Bierbach U., Giaever G. (2012). Comparative chemogenomics to examine the mechanism of action of DNA-targeted platinum-acridine anticancer agents. ACS Chem. Biol..

[36] Kostrhunova H., Malina J., Pickard A.J., Stepankova J., Vojtiskova M., Kasparkova J., Muchova T., Rohlfing M.L., Bierbach U., Brabec V. (2011). Replacement of a thiourea with an amidine group in a monofunctional platinum–acridine antitumor agent. Effect on DNA interactions, DNA adduct recognition and repair. Mol. Pharm..

[37] Martins E.T., Baruah H., Kramarczyk J., Saluta G., Day C.S., Kucera G.L., Bierbach U. (2001). Design, Synthesis, and biological activity of a novel non-cisplatin-type platinum−acridine pharmacophore. J. Med. Chem..

[38] Baruah H., Wright M.W., Bierbach U. (2005). Solution structural study of a DNA duplex containing the Guanine-N7 adduct formed by a cytotoxic platinum−acridine hybrid agent. Biochemistry.

[39] Ma Z., Choudhury J.R., Wright M.W., Day C.S., Saluta G., Kucera G.L., Bierbach U. (2008). A non-cross-linking platinum−acridine agent with potent activity in non-small-cell lung cancer. J. Med. Chem..

[40] Zou T., Liu J., Lum C.T., Ma C., Chan R.C.T., Lok C.N., Kwok W.M., Che C.M. (2014). Luminescent cyclometalated Platinum(II) complex forms emissive intercalating adducts with double-stranded DNA and RNA: Differential emissions and anticancer activities. Angew. Chem. Int. Ed..

[41] Naik A., Rubbiani R., Gasser G., Spingler B. (2014). Visible-light-induced annihilation of tumor cells with platinum–porphyrin conjugates. Angew. Chem..

[42] Finney L., Vogt S., Fukai T., Glesne D. (2009). Copper and angiogenesis: Unravelling a relationship key to cancer progression. Clin. Exp. Pharmacol. Physiol..

[43] Wende C., Lüdtke C., Kulak N. (2014). Copper complexes of N-donor ligands as artificial nucleases. Eur. J. Inorg. Chem..

[44] Brewer G.J., Dick R.D., Grover D.K., LeClaire V., Tseng M., Wicha M., Pienta K., Redman B.G., Jahan T., Sondak V.K. (2000). Treatment of metastatic cancer with tetrathiomolybdate, an anticopper, antiangiogenic agent: Phase I study. Clin. Cancer Res..

[45] Pass H.I., Brewer G.J., Dick R., Carbone M., Merajver S. (2008). A Phase II trial of tetrathiomolybdate after surgery for malignant mesothelioma: Final results. Ann. Thorac. Surg..

[46] Lu J., Sun Q., Li J.L., Jiang L., Gu W., Liu X., Tian J.L., Yan S.P. (2014). Two water-soluble Copper(II) complexes: Synthesis, characterization, DNA cleavage, protein binding activities and in vitro anticancer activity studies. J. Inorg. Biochem..

[47] Liu Y.H., Li A., Shao J., Xie C.Z., Song X.Q., Bao W.G., Xu J.Y. (2016). Four Cu(II) complexes based on antitumor chelators: Synthesis, structure, DNA binding/damage, HSA interaction and enhanced cytotoxicity. Dalton Trans..

[48] Ma Z., Zhang B., Guedes da Silva M.F.C., Silva J., Mendo A.S., Baptista P.V., Fernandes A.R., Pombeiro A.J.L. (2016). Synthesis, Characterization, thermal properties and antiproliferative potential of Copper(II) 4′-phenyl-terpyridine compounds. Dalton Trans..

[49] Molphy Z., Prisecaru A., Slator C., Barron N., McCann M., Colleran J., Chandran D., Gathergood N., Kellett A. (2014). Copper phenanthrene oxidative chemical nucleases. Inorg. Chem..

[50] Gupta A., Lutsenko S. (2009). Human copper transporters: Mechanism, role in human diseases and therapeutic potential. Future Med. Chem..

[51] Palanimuthu D., Shinde S.V., Somasundaram K., Samuelson A.G. (2013). In vitro and in vivo anticancer activity of copper bis(thiosemicarbazone) complexes. J. Med. Chem..

[52] Zhou X.Q., Li Y., Zhang D.Y., Nie Y., Li Z.J., Gu W., Liu X., Tian J.L., Yan S.P. (2016). Copper complexes based on chiral schiff-base ligands: DNA/BSA binding ability, DNA cleavage activity, cytotoxicity and mechanism of apoptosis. Eur. J. Med. Chem..

[53] Lian W.J., Wang X.T., Xie C.Z., Tian H., Song X.Q., Pan H.T., Qiao X., Xu J.Y. (2016). Mixed-ligand Copper(II) schiff base complexes: The role of the co-ligand in DNA binding, DNA cleavage, protein binding and cytotoxicity. Dalton Trans..

[54] Meenongwa A., Brissos R.F., Soikum C., Chaveerach P., Gamez P., Trongpanich Y., Chaveerach U. (2016). Effects of *N*,*N*-heterocyclic ligands on the in vitro cytotoxicity and DNA interactions of Copper(II) chloride complexes from amidino-*O*-methylurea ligands. New J. Chem..

[55] Puckett C.A., Barton J.K. (2007). Methods to explore cellular uptake of ruthenium complexes. J. Am. Chem. Soc..

[56] Howerton B.S., Heidary D.K., Glazer E.C. (2012). Strained ruthenium complexes are potent light-activated anticancer agents. J. Am. Chem. Soc..

[57] Hartinger C.G., Jakupec M.A., Zorbas-Seifried S., Groessl M., Egger A., Berger W., Zorbas H., Dyson P.J., Keppler B.K. (2008). KP1019, a new redox-active anticancer agent—Preclinical development and results of a clinical phase I study in tumor patients. Chem. Biodivers..

[58] Leijen S., Burgers S.A., Baas P., Pluim D., Tibben M., van Werkhoven E., Alessio E., Sava G., Beijnen J.H., Schellens J.H.M. (2015). Phase I/II study with ruthenium compound NAMI-A and gemcitabine in patients with non-small cell lung cancer after first line therapy. Investig. New Drugs.

[59] Hall J.P., Cook D., Morte S.R., McIntyre P., Buchner K., Beer H., Cardin D.J., Brazier J.A., Winter G., Kelly J.M. (2013). X-ray crystal structure of rac-[Ru(phen)2dppz]^2+^ with d(ATGCAT)_2_ shows enantiomer orientations and water ordering. J. Am. Chem. Soc..

[60] Bhat S.S., Revankar V.K., Khan A., Butcher R.J., Thatipamula K. (2015). Supramolecular architecture and photophysical and biological properties of Ruthenium(II) polypyridyl complexes. New J. Chem..

[61] Chen L., Peng F., Li G., Jie X., Cai K.R., Cai C., Zhong Y., Zeng H., Li W., Zhang Z. (2016). The studies on the cytotoxicity in vitro, cellular uptake, cell cycle arrest and apoptosis-inducing properties of ruthenium methylimidazole complex [Ru(MeIm)4(p-cpip)]^2+^. J. Inorg. Biochem..

[62] Mari C., Pierroz V., Rubbiani R., Patra M., Hess J., Spingler B., Oehninger L., Schur J., Ott I., Salassa L. (2014). DNA intercalating Ru^II^ polypyridyl complexes as effective photosensitizers in photodynamic therapy. Chem. Eur. J..

[63] Kaspler P., Lazic S., Forward S., Arenas Y., Mandel A., Lilge L. (2016). A Ruthenium(II) based photosensitizer and transferrin complexes enhance photo-physical properties, cell uptake, and photodynamic therapy safety and efficacy. Photochem. Photobiol. Sci..

[64] Fong J., Kasimova K., Arenas Y., Kaspler P., Lazic S., Mandel A., Lilge L. (2015). A novel class of ruthenium-based photosensitizers effectively kills in vitro cancer cells and in vivo tumors. Photochem. Photobiol. Sci..

[65] Padilla R., Rodriguez-Corrales J.A., Donohoe L.E., Winkel B.S.J., Brewer K.J. (2016). A new class of Ru(II) polyazine agents with potential for photodynamic therapy. Chem. Commun..

[66] Padilla R., Maza W.A., Dominijanni A.J., Winkel B.S.J., Morris A.J., Brewer K.J. (2016). Pushing the limits of structurally-diverse light-harvesting Ru(II) metal-organic chromophores for photodynamic therapy. J. Photochem. Photobiol. A.

[67] Caruso F., Monti E., Matthews J., Rossi M., Gariboldi M.B., Pettinari C., Pettinari R., Marchetti F. (2014). Synthesis, characterization, and antitumor activity of water-soluble (arene)ruthenium(II) derivatives of 1,3-Dimethyl-4-acylpyrazolon-5-ato ligands. First example of Ru(arene)(ligand) antitumor species involving simultaneous Ru–N7(guanine) bonding and ligand intercalation to DNA. Inorg. Chem..

[68] Colina-Vegas L., Villarreal W., Navarro M., de Oliveira C.R., Graminha A.E., Maia P.I.D.S., Deflon V.M., Ferreira A.G., Cominetti M.R., Batista A.A. (2015). Cytotoxicity of Ru(II) piano–stool complexes with chloroquine and chelating ligands against breast and lung tumor cells: Interactions with DNA and BSA. J. Inorg. Biochem..

[69] Liu N., Li X., Huang H., Zhao C., Liao S., Yang C., Liu S., Song W., Lu X., Lan X. (2014). Clinically used antirheumatic agent auranofin is a proteasomal deubiquitinase inhibitor and inhibits tumor growth. Oncotarget.

[70] Zou T., Lum C.T., Lok C.N., Zhang J.J., Che C.M. (2015). Chemical biology of anticancer Gold(III) and Gold(I) complexes. Chem. Soc. Rev..

[71] Nardon C., Fregona D. (2016). Gold(III) complexes in the oncological preclinical arena: From aminoderivatives to peptidomimetics. Curr. Top. Med. Chem..

[72] Rubbiani R., Salassa L., de Almeida A., Casini A., Ott I. (2014). Cytotoxic Gold(I) *N*-heterocyclic carbene complexes with phosphane ligands as potent enzyme inhibitors. ChemMedChem.

[73] Holenya P., Can S., Rubbiani R., Alborzinia H., Junger A., Cheng X., Ott I., Wolfl S. (2014). Detailed analysis of pro-apoptotic signaling and metabolic adaptation ttriggered by a *N*-heterocyclic carbene-gold(I) complex. Metallomics.

[74] Nardon C., Schmitt S.M., Yang H., Zuo J., Fregona D., Dou Q.P. (2014). Gold(III)-dithiocarbamato peptidomimetics in the forefront of the targeted anticancer therapy: Preclinical studies against human breast neoplasia. PLoS ONE.

[75] Akerman K.J., Fagenson A.M., Cyril V., Taylor M., Muller M.T., Akerman M.P., Munro O.Q. (2014). Gold(III) macrocycles: Nucleotide-specific unconventional catalytic inhibitors of human topoisomerase I. J. Am. Chem. Soc..

[76] Meyer A., Oehninger L., Geldmacher Y., Alborzinia H., Wölfl S., Sheldrick W.S., Ott I. (2014). Gold(I) *N*-heterocyclic carbene complexes with naphthalimide ligands as combined thioredoxin reductase inhibitors and DNA intercalators. ChemMedChem.

[77] Liu S., Cao W., Yu L., Zheng W., Li L., Fan C., Chen T. (2013). Zinc(II) complexes containing bis-benzimidazole derivatives as a new class of apoptosis inducers that trigger DNA damage-mediated p53 phosphorylation in cancer cells. Dalton Trans..

[78] Haribabu J., Jeyalakshmi K., Arun Y., Bhuvanesh N.S.P., Perumal P.T., Karvembu R. (2015). Synthesis, DNA/protein binding, molecular docking, DNA cleavage and in vitro anticancer activity of Nickel(II) bis(thiosemicarbazone) complexes. RSC Adv..

[79] Zhang H.R., Liu Y.C., Meng T., Qin Q.P., Tang S.F., Chen Z.F., Zou B.Q., Liu Y.N., Liang H. (2015). Cytotoxicity, DNA binding and cell apoptosis induction of a Zinc(II) complex of HBrQ. Med. Chem. Commun..

[80] Tabrizi L., McArdle P., Erxleben A., Chiniforoshan H. (2015). Nickel(II) and Cobalt(II) complexes of lidocaine: Synthesis, structure and comparative in vitro evaluations of biological perspectives. Eur. J. Med. Chem..

[81] Wani W.A., Baig U., Shreaz S., Shiekh R.A., Iqbal P.F., Jameel E., Ahmad A., Mohd-Setapar S.H., Mushtaque M., Ting Hun L. (2016). Recent advances in iron complexes as potential anticancer agents. New J. Chem..

[82] Yu Y., Gutierrez E., Kovacevic Z., Saletta F., Obeidy P., Rahmanto Y.S., Richardson D.R. (2012). Iron chelators for the treatment of cancer. Curr. Med. Chem..

[83] Del Oliveira A.C., dalSilva E.G., Rocha D.D., Hillard E.A., Pigeon P., Jaouen G., Rodrigues F.A.R., de Abreu F.C., da Rocha Ferreira F., Goulart M.O.F. (2014). Molecular mechanism of action of 2-Ferrocenyl-1,1-diphenylbut-1-ene on HL-60 leukemia cells. ChemMedChem.

